# Reversal learning paradigm reveals deficits in cognitive flexibility in the *Fmr1 *knockout male mouse

**DOI:** 10.12688/f1000research.14969.1

**Published:** 2018-06-07

**Authors:** Suzanne O. Nolan, Joaquin N. Lugo

**Affiliations:** 1 Department of Psychology and Neuroscience, Baylor University, Waco, TX, 76798, USA; 2 Institute of Biomedical Studies, Baylor University, Waco, TX, 76798, USA; 3 Department of Biology, Baylor University, Waco, TX, 76798, USA

**Keywords:** Cognitive flexibility, Autism, Morris water maze, Prefrontal cortex, FMRP

## Abstract

**Background: **Loss of *FMR1 *is associated with Fragile X syndrome, amongst the most prevalent inherited intellectual disability. Despite extensive research in this area, previous studies have failed to detect consistent evidence of cognitive impairments in the Morris water maze (MWM) task in the *Fmr1 *knockout (KO) mouse. However, few studies have examined cognitive flexibility in a reversal form of the MWM task, which may illuminate subtle learning deficits.

**Methods:** Adult male *Fmr1 *wildtype (WT) and KO mice were bred and tested in the MWM reversal paradigm. The testing paradigm consisted of two blocks per day, with 4 trials per block to locate a hidden platform. After the last trials on the fourth day of testing, the animals were given a probe trial with the platform removed. The following week, the location of the platform was switched to the opposite quadrant and the animals received 2 more days of testing, with 4 blocks in total.

**Results:** As expected, *Fmr1 *KO mice did not display a learning deficit during the acquisition phase, *F _genotype _*(1, 24) = 0.034, *p* = 0.854, and performed similarly on the probe trial, *F _genotype _*(1, 23) = 0.024, *p* = 0.877. However, during the reversal phase of learning, *Fmr1 *KO mice showed deficits in their ability to learn the new location of the platform, *F _genotype _*(1, 23) = 3.93, *p* = 0.059. Further independent samples t-testing revealed that KO animals displayed significantly higher latency to reach the hidden platform during the third trial, *t*(23) = -2.96, *p *< 0.01.

**Conclusions: **While previous studies have not demonstrated deficits in spatial memory in the *Fmr1 *KO model, it is possible that the acquisition phase of the task is less sensitive to deficits in learning. Future studies using this model to evaluate therapeutic interventions should consider utilizing the MWM reversal paradigm.

## Introduction

Fragile X syndrome (FXS) is a neurodevelopmental disorder, caused by a trinucleotide expansion mutation in the *FMR1* gene, and is also one of the most prevalent inherited forms of intellectual disability ^1^. FXS is often modeled using the *Fmr1* knockout (KO) mouse, which can be characterized by several behavioral phenotypes, including alterations in sociability and deficits in fear memory ^2– 4^. Aside from deficits in spatial and non-spatial learning, one understudied facet of intellectual disability is the ability to incorporate new information into existing learning, termed cognitive flexibility. Cognitive flexibility can be studied in rodents using a variant of the Morris water maze (MWM) paradigm ^5, 6^. In the MWM reversal paradigm, the location of the hidden platform is moved, and the latency to adjust to the new location is measured. As expected, several reports find evidence of impairments in reversal learning in the *Fmr1* KO mouse across multiple strains, the C57BL/6J backcrossed strain ^7, 8^ and the albino C57BL/6J background ^2^. However, other studies have been unable to replicate these findings, and further investigation points to the possibility of background strain differences ^9^. Previous reports have not detected any impairments in reversal learning in the FVB.129 strain ^10^. However, it may be that this paradigm is perhaps even more sensitive to methodological differences. The current study adds to this literature by using the FVB.129 strain in a previously utilized paradigm.

## Methods

### Animals

Male *Fmr1* ^+/+^ and female *Fmr1* ^+/-^ FVB.129P2-Pde6b+Tyrc-ch Fmr1tm1Cgr/J (Stock No: 004624, The Jackson Laboratory, Bar Harbor, ME, USA) mice were used as breeders (9 total breeding pairs) to produce the following groups: male WT and male KO pups. Breeding pairs were of the following groupings: WT Female/WT Male (n = 2), KO Female/KO Male (n = 5), WT Female/KO Male (n = 2). Genotype was determined from toe clippings taken prior to age postnatal day (PD) 12 (Mouse Genotype, Escondido, CA, USA). The final sample sizes were as follows: n _male WT_ = 10, n _male KO_ = 16. Target samples sizes (n = 10) were calculated *a priori* using a power calculation in G*Power 3.1 with the following parameters: f = 0.50 (large effect), α = 0.05, power (1 – β) = 0.80, for the F family of tests with two groups and 8 repeated measures (trials). All pups were housed in individual cages (Allentown Caging PC7115HT, Allentown, PA, USA), filled with sani-chip bedding (7090 Teklad, Envigo, Somerset, NJ, USA). Prior to weaning on PD21, pups were housed with parents (1 male and 2 females) and littermates (up to 12 pups). Following weaning, subjects were housed with mixed genotype littermates, no more than 5 to a cage. The light cycle was kept at 12 hr. light, and the colony room was kept at an ambient temperature of 22° C. Animals had *ad libitum* access to food and water. All procedures performed were in accordance with *Baylor University Institutional Animal Care and Use Committee* (Animal Assurance Number A3948-01), as well as the *National Institutes of Health Guidelines for the Care and Use of Laboratory Animals*. All efforts were made to ameliorate any stress and harm to the animals, specifically by habituating animals to the testing apparatus and room prior to trial recordings.

### Morris water maze

**Figure 1.**
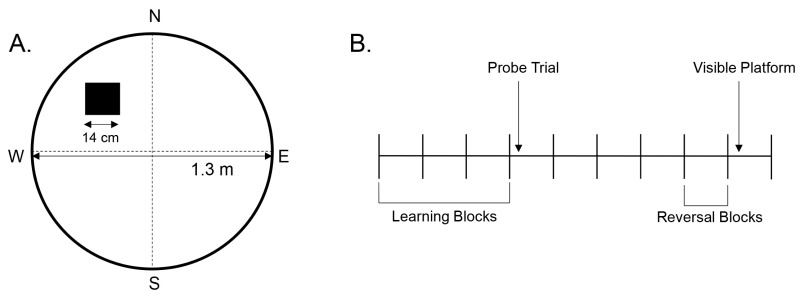
Overview of the testing paradigm. **A**. An overview of the set-up of the testing arena. **B**. An overview of the progression of testing days.

All behavioral testing was conducted during the light cycle, specifically between 8 am and 5 pm. The methods for the current study were adapted as closely as possible from earlier studies of this behavior in the *Fmr1* KO mouse (represented in Figure 1) ^9^. Briefly, a 1.3 m diameter white pool was filled with water and made opaque through the addition of non-toxic white paint (Item LT3010, S&S Worldwide, Connecticut). The hidden platform measured 14.5 cm × 14.5 cm and was submerged approximately 2 cm below the water level. The testing paradigm consisted of two blocks per day for 4 days, with 4 trials in each block, for each mouse to test the ability to locate a hidden platform. The mice were habituated to the testing room in their holding cages for 30 minutes prior to the onset of testing. The amount of time spent in each quadrant for each trial was recorded with a ceiling-mounted video camera (Ganz YCH-02, Cary, NC, USA), and analyzed using automated tracking software (Ethovision XT 6, Noldus, Wageningen, Netherlands). After the last trial on the fourth day of testing was completed, the animals were given a probe trial. The probe trial involved removing the platform and allowing the subjects to explore the maze for 60 seconds. During the probe trial, the number of times the animal crossed the location of the hidden platform and the duration of time in each quadrant was calculated. Testing resumed on day 8 after a 3-day rest period. On day 8, the platform was placed in the opposite quadrant from the previous location that housed the hidden platform. Testing progressed as with the initial acquisition phase, with 2 blocks per day for 2 days. On the final day of testing, a visible platform was used to evaluate visual performance as well as swim speed. The visible platform was a two-tiered platform similar to the initial platform, with a second higher tier platform that extended 9.5 cm above the lower platform, allowing the animal to see the platform. The differences in methodology from the cited source were as follows: only four days of acquisition were conducted and the testing paradigm was lengthened to account for a consolidation period between the learning and reversal trials (See Figure 1 for a description of the testing paradigm). One KO animal was excluded from analysis due to a seizure during this task.

### Statistical analysis

 Statistical analysis was performed in the form of a one-way analysis of variance (ANOVA) with one between-subjects factor (Genotype [wildtype, knockout]) and one within-subjects factor (Trial). All data were analyzed using GraphPad Prism Software 7.0 (San Diego, CA, USA) or IBM SPSS Statistics 23 (Aramonk, NY, USA).

## Results

### *Fmr1* KO mice show no impairment in acquisition of spatial memory

**Figure 2.**
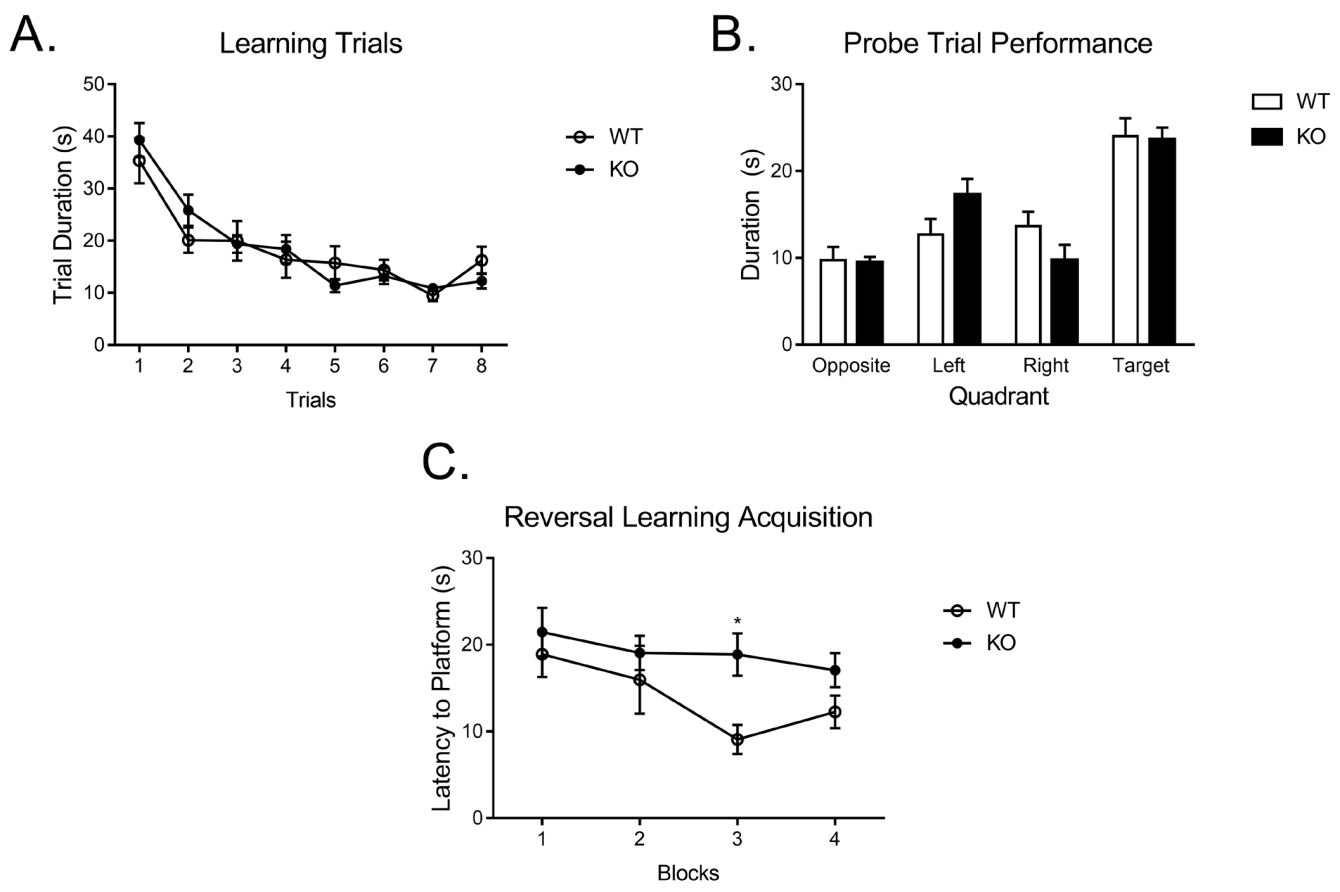
Performance in the Morris water maze in *Fmr1* knockout males. **A**. *Fmr1* knockout males show no deficits in acquisition in performance. **B**. Performance during the probe trial is not impaired in the *Fmr1* knockout males. **C**. When subjected to a reversal learning paradigm, *Fmr1* knockout mice display increased latency to the new platform location, demonstrating deficits in cognitive flexibility. Knockout – KO, Wild type - WT.

To investigate the effect of genotype on hippocampal spatial memory, animals were tested in the MWM paradigm ( Dataset 1). During the 8 blocks of learning trials ( Figure 2A), there was a significant within-subjects effect of trial for latency to reach the platform, *F*(3.56, 85.47) = 30.15, *p* < .0005. Trial results did not interact significantly with genotype, *F*(3.56, 85.47) = 1.33, *p* = 0.24, suggesting both groups learned the location of the platform similarly. Between-subjects analyses indicated no effect of genotype, *F*(1, 24) = 0.03, *p* = 0.85. Further independent samples t-testing revealed no differences between WT and KO at any of the 8 different trials, *p* > 0.05.

For the probe trial ( Dataset 2), as expected, male KO mice demonstrated similar time spent in the target quadrant, *F*(1,23) = 0.02, *p* = 0.88 ( Figure 2B), compared to male wildtype (WT). Further independent samples t-testing revealed no differences in duration in any of the quadrants, *p* > 0.05.

### Loss of *Fmr1* impairs ability to update existing learning with new platform location

The week following the initial learning trials, animals were tested in the reversal learning paradigm ( Dataset 3). During the 4 blocks of learning trials ( Figure 2C), a one-way ANOVA with repeated measures revealed a significant within-subjects effect of trial, *F*(3, 69) = 3.8, *p* < 0.05. Trial results did not interact significantly with genotype, *F*(3, 69) = 1.28, *p* = 0.29. However, between-subjects analyses indicated a marginal effect of genotype, *F*(1, 23) = 3.93, *p* = 0.059 ( Figure 2C). Further independent samples t-testing revealed that KO animals displayed significantly higher latency to reach the hidden platform during the third trial, *t*(23) = -2.96, *p* < 0.01. Together, these results demonstrate that *Fmr1* KO males demonstrate decreased learning and altogether a lack of cognitive flexibility across all trials of the MWM reversal task.

### Impairments were not due to deficits in vision or motor capabilities

**Figure 3.**
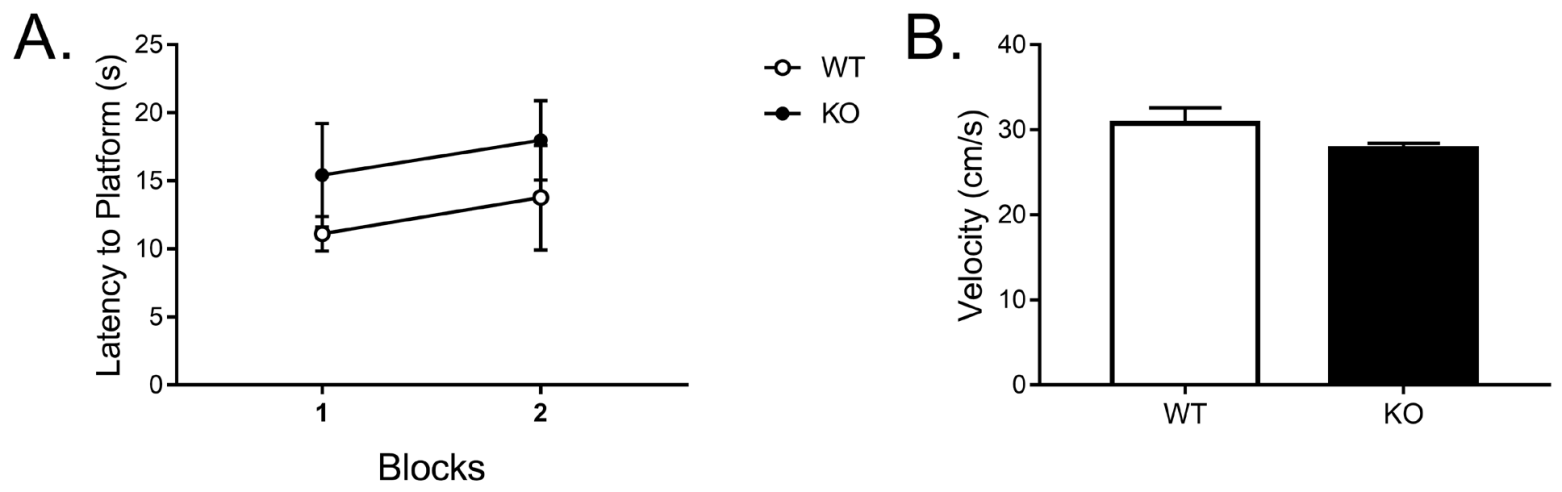
Performance in the Morris water maze visible trials. **A**. *Fmr1* knockout males showed no deficits in latency to the platform across the two blocks of the visible platform test. **B**. *Fmr1* knockout males showed no deficits in swim speed across the visible platform test. Knockout – KO, Wild type - WT.

Visible platform information was also assessed to ensure differences were not due to deficits in vision ( Dataset 4). Results were analyzed using a repeated-measures ANOVA across the four visible platform trials on latency to the platform across the two blocks of trials. Results indicated no effect of block, *F*(1, 24) = 1.341, *p* = 0.26, nor an interaction of block and genotype, *F*(1, 24) = 0.0005, *p* = 0.98. There was not a significant effect of genotype on latency to the platform during these trials either, *F*(1, 24) = 0.98, *p* = 0.33 ( Figure 3A). Altogether, these data suggest that differences in latency to the platform could not be attributed to deficits in visual perception in the *Fmr1* KO male mouse. Moreover, differences in latency to the platform on the previous trials could not be attributed to impairments in swimming abilities, as no differences in swim speed were detected during the visible trials, *t*(11.17) = 1.526, *p* = 0.16 ( Figure 3B).

Learning Trial Data This datasheet contains the raw data exported from the Ethovision program (Columns A – G) as well as the transformed dataset that was used for analysis for the learning trials (Columns J – T) Click here for additional data file. Copyright: © 2018 Nolan SO and Lugo JN 2018 Data associated with the article are available under the terms of the Creative Commons Zero "No rights reserved" data waiver (CC0 1.0 Public domain dedication).

Probe Trial Data This datasheet contains the raw data exported from the Ethovision program (Columns A - AB) as well as the transformed dataset that was used for analysis for the probe trial (Columns AC – AI) Click here for additional data file. Copyright: © 2018 Nolan SO and Lugo JN 2018 Data associated with the article are available under the terms of the Creative Commons Zero "No rights reserved" data waiver (CC0 1.0 Public domain dedication).

Reversal Trial Data This datasheet contains the raw data exported from the Ethovision program (Columns A – G) as well as the transformed dataset that was used for analysis for the reversal trials (Columns J – O) Click here for additional data file. Copyright: © 2018 Nolan SO and Lugo JN 2018 Data associated with the article are available under the terms of the Creative Commons Zero "No rights reserved" data waiver (CC0 1.0 Public domain dedication).

Visible Platform Data This datasheet contains the raw data exported from the Ethovision program (Columns A – H) as well as the transformed dataset that was used for analysis of the visible platform trials (Columns K – S) Click here for additional data file. Copyright: © 2018 Nolan SO and Lugo JN 2018 Data associated with the article are available under the terms of the Creative Commons Zero "No rights reserved" data waiver (CC0 1.0 Public domain dedication).

## Discussion

 As expected, deletion of *Fmr1* did not impact initial spatial learning in the MWM. The current study did, however, demonstrate impairments in cognitive flexibility in the *Fmr1* KO. As previously mentioned, other studies have not before detected such changes, and this discrepancy could be attributed to methodological differences ^10^. In the aforementioned study, training occurred over 8 days, with only 3 training trials per day, and the reversal paradigm consisted of 4 days, with 3 trials per day. Furthermore, in support of our findings, deficits in long-term potentiation in the prefrontal cortex have been demonstrated in the *Fmr1* KO mouse, the area on which the ability to adapt to a new location in this task is dependent on 11– 13. Moreover, this ability to adapt to a new location is mediated through multi-synaptic connections between the hippocampus and the prefrontal cortex ^13^. This proposed mechanism further supports our findings of no change to the initial spatial learning phase, as lesions to this area did not impact initial learning performance in spatial navigation ^11^.

The current study provides preliminary evidence that could be applied to tease apart subtle differences between the male and female *Fmr1* KO phenotype, which has been difficult to conclusively evaluate. Future studies should expand upon these findings in females, as many studies have demonstrated a sex-specific effect of loss of *Fmr1* on behavior (discussed in a recent review ^14^) ^2– 4, 15^. Overall, this study corroborates and extends previous evidence of impaired cognitive flexibility in the male *Fmr1* KO mouse.

## Data availability

The data referenced by this article are under copyright with the following copyright statement: Copyright: © 2018 Nolan SO and Lugo JN

Data associated with the article are available under the terms of the Creative Commons Zero "No rights reserved" data waiver (CC0 1.0 Public domain dedication). 

**Dataset 1– “Learning Trial Data – CSV.csv”**

This datasheet contains the raw data exported from the Ethovision program (Columns A – G) as well as the transformed dataset that was used for analysis for the learning trials (Columns J – T). http://dx.doi.org/10.5256/f1000research.14969.d206068 ^16^

**Dataset 2 – “Probe Trial Data – CSV.csv”**

This datasheet contains the raw data exported from the Ethovision program (Columns A - AB) as well as the transformed dataset that was used for analysis for the probe trial (Columns AC – AI). http://dx.doi.org/10.5256/f1000research.14969.d206069 ^17^

**Dataset 3 – “Reversal Trial Data – CSV.csv”**

This datasheet contains the raw data exported from the Ethovision program (Columns A – G) as well as the transformed dataset that was used for analysis for the reversal trials (Columns J – O). http://dx.doi.org/10.5256/f1000research.14969.d206076 ^18^

**Dataset 4 – “Visible Platform Data – CSV.csv”**

This datasheet contains the raw data exported from the Ethovision program (Columns A – H) as well as the transformed dataset that was used for analysis of the visible platform trials (Columns K – S). http://dx.doi.org/10.5256/f1000research.14969.d206077 ^19^
