# Supplementary material for: Reversal learning paradigm reveals deficits in cognitive flexibility in the Fmr1 knockout male mouse
Source: F1000Res. 2018 Jun 7;7:711. [Version 1] doi: 10.12688/f1000research.14969.1 (PMC6051189; doi:10.12688/f1000research.14969.1)
Supplement: Peer review history

# Peer review: Referee response for version 1

**DOI:** 10.5256/f1000research.16297.r34793

**Published:** 2018-07-16

**Authors:** Lisa Monteggia

**Affiliations:** 1 Department of Neuroscience, University of Texas Southwestern Medical Center, Dallas, TX, USA

The current study examines male Fmr1 knockout mice in the Morris Water maze.  This group focuses on the FVB strain  as previous work has shown background strain differences can impact the phenotype of Fmr1 knockouts.  The authors show that the male Fmr1 knockout mice have no impairment in acquisition of spatial memory but do show differences in the reversal learning paradigm that are not attributed to vision or motor differences.  In the reversal learning paradigm, the authors show that during the 4 blocks of reversal learning the Fmr1 KOs show a rather stable latency to find the platform in contrast to the WT mice that show the expected decrease to reach the platform with increasing trials.  The authors highlight the finding that male Fmr1 knockout mice have deficits in reversal learning as assessed in the Morris water maze but not in acquisition, demonstrating a link to a specific form of learning in this paradigm.  The study is straightforward and the data clearly presented. 

 A couple of comments the authors should further clarify:

 The authors should elaborate on the breeding scheme as they are not directly comparing littermate controls. 

 Based on the authors discussion point regarding potential sex differences between male and female Fmr1 KO mice, is there data to suggest differences in cognitive flexibility between the males and female KOs?

I have read this submission. I believe that I have an appropriate level of expertise to confirm that it is of an acceptable scientific standard.

# Peer review: Referee response for version 1

**DOI:** 10.5256/f1000research.16297.r34795

**Published:** 2018-07-02

**Authors:** Andre Fenton

**Affiliations:** 1 Center for Neural Science, New York University, New York, NY, USA

I am afraid that I did not find this manuscript and the results to be compelling.

 The experimental design is straightforward but the analysis is questionable and the report is not as scholarly as it should be.

 Statistics are used questionably. 1) t tests are used for post-hoc comparisons, when they are not justified, For example, post-hoc t tests are performed after ANOVA has failed to find relevant differences. 2) Furthermore, the alpha level for post-hoc t tests seem  not to be corrected for multiple comparisons. The reported significant difference on trial 3 may not maintain after correcting alpha for multiple comparisons 0.05/4, although it might - a reader can’t know. Regardless, what is the justification for the t tests after the ANOVA results were not significant?

 Dataset 1: Given no effects of genotype or interaction in the ANOVA, what justifies data snooping as follows: "Further independent samples t-testing revealed no differences between WT and KO at any of the 8 different trials, p > 0.05.” ? 

 Dataset 2: Given the ANOVA was not significant for a 1-way ANOVA, there is no justification for a t test and in fact it is numerically equivalent to the F-test, so even more so not warranted.

 "Further independent samples t-testing revealed no differences in duration in any of the quadrants, p > 0.05.”

 The manuscript states that "Fmr1 KO mice showed deficits in their ability to learn the new location of the platform, Fgenotype (1, 23) = 3.93, p = 0.059.” Why is p= 0.059 indicative of a deficit?

 The Introduction is written as if the water maze is the only way to test spatial memory and cognitive flexibility, when of course it is not.

 The Discussion states "The current study did, however, demonstrate impairments in cognitive flexibility in the Fmr1 KO. As previously mentioned, other studies have not before detected such changes“ Remarkably, the authors fail to consider the work from the Fenton lab which explicitly has investigated cognitive flexibility in Fmr1 KO mice (Radwan et al., 2015 Neurobiol. Dis.; Dvorak et al., 2018, PLoS Biol ^1^.) along with electrophysiological correlates of the ability.

 Something is wrong with the initial statement: "Male Fmr1+/+ and female Fmr1+/- FVB.129P2-Pde6b+Tyrc-ch Fmr1tm1Cgr/J (Stock No: 004624, The Jackson Laboratory, Bar Harbor, ME, USA) mice were used as breeders (9 total breeding pairs) to produce the following groups:” because WT mice cannot contribute to breeding involving a KO male.

 Fig 1B should have the days indicated, as well as the rest period.

 Fig. 2C shows poor learning even in WT, despite the significant effect of trial. Could the paradigm simply not be robust in mice? There is a literature on this possibility, see Wolfer et al., 1998

 News Physiol Sci ^2^.).

 What justification is there for the assertion that "Moreover, this ability to adapt to a new location is mediated through multi-synaptic connections between the hippocampus and the prefrontal cortex13.”?

 What is the mechanism that is referenced by the statement "This proposed mechanism further supports our findings of no change to the initial spatial learning phase, as lesions to this area did not impact initial learning performance in spatial navigation11.”?

 The final statement seems untrue given that the authors claim to find a reversal deficit but say there had not been one detected previously "Overall, this study corroborates and extends previous evidence of impaired cognitive flexibility in the male Fmr1 KO mouse.”

 What is "t-testing" should this not simply be stated as "t tests” ?

I have read this submission. I believe that I have an appropriate level of expertise to confirm that it is of an acceptable scientific standard, however I have significant reservations, as outlined above.

## References

[1] DvorakDRadwanBSparksFTTalbotZNFentonAA: Control of recollection by slow gamma dominating mid-frequency gamma in hippocampus CA1. *PLoS Biol*.2018;16(1) : 10.1371/journal.pbio.2003354 e2003354 10.1371/journal.pbio.2003354 29346381PMC5790293

[2] WolferDPStagljar-BozicevicMErringtonMLLippHP: Spatial Memory and Learning in Transgenic Mice: Fact or Artifact?. *News Physiol Sci*.1998;13:118-123 1139077410.1152/physiologyonline.1998.13.3.118

# Peer review: Referee response for version 1

**DOI:** 10.5256/f1000research.16297.r34794

**Published:** 2018-06-20

**Authors:** Laura Smith

**Affiliations:** 1 Department of Neuroscience & Experimental Therapeutics, Texas A&M Health Science Center, Bryan, TX, USA

The study authored by Nolan and Lugo investigates the ability of *Fmr1* KO mice on the FVB.129 background to demonstrate proper reversal learning in the Morris water maze (MWM). Background strain appears to make a large difference in observed cognitive deficits in mice lacking expression of the *Fmr1* gene, and this study makes a valuable contribution to the literature by assessing MWM reversal learning in *Fmr1* KO mice on the pure FVB background.

 Suggested revisions:

1. The authors refer to papers showing impaired reversal learning in *Fmr1* KO mice on the *C57BL/6J* strain and cite D’Hooge et al., 1997 ^1^ and Kooy et al., 1996 ^2^ However, in each of these studies the authors specify that only pigmented offspring were selected for testing, suggesting that these mouse lines were not fully backcrossed to C57BL/6J. Of note, stem cells used in the creation of transgenic mice have often been derived from the 129 strain. In any case, the authors should likely revisit this statement, and instead may wish to report Paradee et al. (1999) ^3^ which used fully backcrossed C57BL/6 KO mice.
2. The statement that “few studies have examined cognitive flexibility in a reversal form of the MWM task” (presumably in *Fmr1* KO mice) seems inaccurate, as most studies using MWM to assess *Fmr1* KO mice actually include this task (Paradee et al., 1999; Kooy et al., 1996; D’Hooge et al., 1997; The Dutch-Belgian Fragile X Consortium paper, 1994 ^4^; Baker et al., 2010) ^5^. However, as the authors state, it had not been properly assessed on the FVB background.
3. The abstract conclusion says that “previous studies have not demonstrated deficits in spatial memory in the *Fmr1* KO model.” However, multiple previous assessments of *Fmr1* KO mice in the MWM have shown minor acquisition differences compared to WT mice (e.g., Paradee et al., 1999; Kooy et al., 1996, *Am J Med Genet*; The Dutch-Belgian Fragile X Consortium paper, 1994).  Please consider these and also deficits observed in the plus-shaped water maze (e.g., Dobkin et al., 2000 ^6^; Van Dam et al., 2000 ^7^) and radial arm maze (Mineur et al., 2002 ^8^) to perhaps soften this statement appropriately.
4. Consider changing “disability” to “disabilities” in first sentence of the abstract.
5. Please add more detail about the Morris water maze task. For instance, did mice receive consecutive trials within each block? Was entry quadrant varied or static over each mouse’s trials within (and across) blocks? Was the temperature of the water controlled? What were the visual cues, where were they positioned with respect to each quadrant, and were they proximal or distal? If animals were artificially colored for detection in the maze, please specify. Were animals dried and/or warmed between or after trials?
6. It may be helpful to label days on Fig. 1B.
7. In the first Results paragraph-- in the absence of a significant main effect or interaction involving genotype, further independent samples t-testing between WT and KO should probably not be reported.
8. In the Discussion, the authors point to deficits in *Fmr1* KO prefrontal cortex LTP with regard to the observed reversal learning deficit, which is fine. However, using this same logic to claim support for *lack *of observed differences in acquisition, based on findings that PFC is not relevant to acquisition, is somewhat flawed. It may be more appropriate (e.g., in the sentence regarding PFC and learning a new location) to simply acknowledge that "PFC is not required for acquisition" and allow the reader to intuit the point (that seems to be intended) more subtly. If this topic will be discussed, other brain regions likely involved in MWM acquisition (e.g., hippocampus), and that also show significant plasticity-related variations in *Fmr1* KO compared to WT mice, should be included.
9. The authors’ point that this investigation should be made in females is well-received. However, it is not immediately clear how these data in male *Fmr1* KO mice provide “preliminary evidence that could be applied to tease apart subtle differences between the male and female *Fmr1* KO phenotype.” Perhaps this point can be further clarified or removed?
10. In the attached data sets, it is not clear why a few animal IDs are repeated. Specifically, please see the “Learning trial” set, and the “transformed” data on the right-hand side of the screen. Subjects 124 and 134 are listed twice, but all other IDs appear once. Also, a little more explanation, either directly on the data sheets or elsewhere in the manuscript, for the trial names, etc. seen here may be beneficial to users of these data.

I have read this submission. I believe that I have an appropriate level of expertise to confirm that it is of an acceptable scientific standard, however I have significant reservations, as outlined above.

## References

[1] D'HoogeRNagelsGFranckFBakkerCEReyniersEStormKKooyRFOostraBAWillemsPJDe DeynPP: Mildly impaired water maze performance in male Fmr1 knockout mice. *Neuroscience*.1997;76(2) :367-76 901532210.1016/s0306-4522(96)00224-2

[2] KooyRD'HoogeRReyniersEBakkerCNagelsGDe BoulleKStormKClinckeGDe DeynPOostraBWillemsP: Transgenic mouse model for the fragile X syndrome. *American Journal of Medical Genetics*.1996;64(2) : 10.1002/(SICI)1096-8628(19960809)64:2<241::AID-AJMG1>3.0.CO;2-X 241-245 10.1002/(SICI)1096-8628(19960809)64:2<241::AID-AJMG1>3.0.CO;2-X 8844056

[3] ParadeeWMelikianHERasmussenDLKennesonAConnPJWarrenST: Fragile X mouse: strain effects of knockout phenotype and evidence suggesting deficient amygdala function. *Neuroscience*.1999;94(1) :185-92 1061350810.1016/s0306-4522(99)00285-7

[4] Fmr1 knockout mice: a model to study fragile X mental retardation. The Dutch-Belgian Fragile X Consortium. *Cell*.1994;78(1) :23-33 8033209

[5] BakerKBWraySPRitterRMasonSLanthornTHSavelievaKV: Male and female Fmr1 knockout mice on C57 albino background exhibit spatial learning and memory impairments. *Genes Brain Behav*.2010;9(6) : 10.1111/j.1601-183X.2010.00585.x 562-74 10.1111/j.1601-183X.2010.00585.x 20398059

[6] DobkinCRabeADumasREl IdrissiAHaubenstockHBrownWT: Fmr1 knockout mouse has a distinctive strain-specific learning impairment. *Neuroscience*.2000;100(2) :423-9 1100818010.1016/s0306-4522(00)00292-x

[7] Van DamDD'HoogeRHaubenEReyniersEGantoisIBakkerCEOostraBAKooyRFDe DeynPP: Spatial learning, contextual fear conditioning and conditioned emotional response in Fmr1 knockout mice. *Behav Brain Res*.2000;117(1-2) :127-36 1109976610.1016/s0166-4328(00)00296-5

[8] MineurYSSluyterFde WitSOostraBACrusioWE: Behavioral and neuroanatomical characterization of the Fmr1 knockout mouse. *Hippocampus*.2002;12(1) : 10.1002/hipo.10005 39-46 10.1002/hipo.10005 11918286
